# Continuous glucose monitoring metrics based clustering in people living with type 1 diabetes identifies phenotypes associated with higher inflammation, metabolic dysfunction-associated steatotic liver disease risk, and lower insulin sensitivity

**DOI:** 10.1186/s13098-026-02191-3

**Published:** 2026-05-25

**Authors:** Aleksejs Fedulovs, Natalia Paramonova, Leonora Pahirko, Una Riekstina, Kaspars Jekabsons, Jelizaveta Sokolovska

**Affiliations:** 1 Faculty of Medicine and Life Sciences, Department of Clinical and Personalized Medicine, Laboratory for Personalized Medicine, University of Latvia, Jelgavas Street 3, Riga, 1004 LV Latvia; 2 Faculty of Medicine and Life Sciences, Department of Pharmaceutical Sciences, University of Latvia, Jelgavas Street 1, Riga, 1004 LV Latvia; 3 Faculty of Science and Technology, University of Latvia, Jelgavas Street 3, Riga, 1004 LV Latvia

**Keywords:** Type 1 diabetes, Continuous glucose monitoring, Metabolic dysfunction-associated steatotic liver disease, Insulin resistance, Intestinal permeability, Low-grade inflammation

## Abstract

**Aims:**

Continuous glucose monitoring (CGM) enables scalable digital phenotyping by capturing detailed daily glucose profiles and has been shown to improve glycaemic control in individuals with type 1 diabetes (T1D). We hypothesized that clustering CGM-derived metrics could identify distinct glycemic phenotypes associated with increased insulin resistance, systemic inflammation, and risk of metabolic derangements, with potential implications for optimization of clinical care.

**Materials and methods:**

This cross-sectional study applied a hierarchical clustering approach to stratify 75 individuals with T1D based on CGM metrics obtained over 14 days (Libre ProIQ). Clustering was performed using Ward’s minimum variance linkage and Euclidean distance on six CGM metrics (average glucose, coefficient of variance, time above range, time below range, time in range, and frequency of low glucose events). The resulting groups, poorly controlled diabetes (PCD) and moderately controlled diabetes (MCD), were compared for insulin resistance markers (estimated glucose disposal rate, eGDR), metabolic dysfunction-associated steatotic liver disease (MASLD) risk markers (fatty liver index, FLI; hepatic steatosis index, HSI), and inflammation and endotoxaemia markers.

**Results:**

Compared to MCD, subjects in the PCD group had significantly higher insulin resistance (eGDR: 4.4 vs. 7.3 mg/kg/min, *p* = 0.001), elevated liver enzymes (ALT: 26 vs. 20 U/L, *p* = 0.005; AST: 28 vs. 23 U/L, *p* = 0.049), increased MASLD risk markers (HSI: 37 vs. 35, *p* = 0.033; FLI: 29 vs. 17, *p* = 0.034), and higher systemic inflammation (CRP: 1.82 vs. 0.81 mg/L, *p* = 0.020; lipopolysaccharide-binding protein (LBP): 11.7 vs. 8.4 EU/mL, *p* = 0.024). Time above range correlated positively with liver and inflammatory markers, while time in range correlated negatively. Mediation analysis suggested that FLI partially mediated the effect of poor glycemic control on inflammation.

**Conclusions:**

In this cross-sectional cohort of adults with T1D, poorer CGM-derived glycaemic control was associated with higher systemic inflammation and higher surrogate-marker-defined MASLD risk.

**Supplementary Information:**

The online version contains supplementary material available at 10.1186/s13098-026-02191-3.

## Introduction

Type 1 diabetes (T1D) is a chronic autoimmune disorder characterized by insulin deficiency, leading to dysregulated glucose homeostasis and increased risk of both acute and chronic complications. Continuous glucose monitoring (CGM) allows detailed tracking of glycemic variability for better treatment outcomes in T1D [1–3]. CGM provides comprehensive data on glucose fluctuations, facilitating the assessment of time spent in the recommended glycaemic range (TIR), time above range (TAR), and time below range (TBR), and glucose variability (GV) among other metrics, for characterization of the glycaemic control, and treatment optimization [4].

Current CGM report provides limited insight into possible associated conditions. However emerging evidence indicates that clustering CGM data may help identify patient subgroups with distinct clinical risks. For example, Kovatchev et al. identified a total of 32 clusters among 204,710 daily CGM profiles in healthy, T1D and type 2 diabetes patients on different treatments, for different applications, including data interpretation, algorithmic approaches to treatment, pattern recognition, and tracking disease progression [5]. Song et al. used Riemannian manifold-based geometric clustering to develop a method linking CGM metrics to long-term glucose control data [6]. CGM data of more than 600 subjects with T1D were analyzed by Discriminative Dimensionality Reduction with Trees method on top of traditional clustering, allowing to identify seven glycaemic phenotypes differing in socioeconomic factors, cardiovascular risk indicators, retinopathy and treatment strategy [7]. In addition, clustering of CGM data based on GV level helped to identify individuals with increased pro-thrombotic biomarkers [8].

The prevalence of overweight, obesity and metabolic-dysfunction-associated steatotic liver disease (MASLD) is increasing in T1D [9, 10], both as a result of improved insulin treatment and as a consequence of “western” diet and sedentary lifestyle spreading globally [11, 12]. It is believed that subcutaneous insulin replacement therapy can contribute to insulin resistance and ectopic fat deposition via relative insulin deficiency in the portal vein and hyperinsulinization of peripheral tissues [11]. Moreover, research data indicate that MASLD and metabolic syndrome are associated with low-grade inflammation and endotoxaemia in T1D [13], which can further worsen the glycaemic control [14, 15] and increase the risk of cardiovascular complications [16–19].

Therefore, timely identification of people with T1D who are at risk of MASLD and low-grade inflammation is important for improving clinical outcomes. In this respect, CGM can be considered not only as a tool for meticulous glucose control, but also as a source of continuous biomonitoring for identification of physiological processes associated with glucose fluctuations. Recent data indicate some CGM parameters can be associated with MASLD in T1D [20, 21], higher levels of low-grade inflammation [22], insulin resistance as assessed by estimated glucose disposal rate (eGDR) [23] and progressive diabetic kidney disease in T1D [24]. This means that CGM might help clinicians to identify subjects with less favorable metabolic and inflammatory profiles and implement prevention and treatment strategies for these conditions in a timely manner. However, to our knowledge, studies utilizing a clustering approach to CGM data for identifying increased levels of inflammation, endotoxaemia and MASLD risk in T1D are currently lacking.

In this study, we hypothesised that clustering of CGM metrics could stratify adults with T1D into groups with distinct metabolic and inflammatory profiles, thereby providing additional insight into associations between glycaemic control and variability, systemic inflammation, and MASLD risk. Our findings suggest that CGM-derived phenotyping may have potential as a biomonitoring approach that extends beyond conventional assessment of glycaemic control and may help identify individuals with less favourable metabolic and inflammatory profiles in T1D.

## Materials and methods

### Study subjects, recruitment, clinical definitions and surrogate markers of MASLD and insulin sensitivity

This study is part of the longitudinal LatDiane study, initiated in 2013, and conducted within the international InterDiane consortium. LatDiane recruits adult patients with clinically defined T1D diagnosed before the age of 40 years, who initiated insulin therapy within one year of diagnosis and had C-peptide levels below 0.3 nmol/L. Although T1D-specific autoantibody data were not available in the present study, the utilized inclusion criteria allow to reduce the likelihood of including individuals with other diabetes types. Patients with chronic kidney disease unrelated to diabetic kidney disease (DKD) are excluded, based on medical record evaluation [25]. The study protocol for the overall LatDiane study and the sub-study reported herein have received approvals from the Latvian Central Ethics Committee (Riga, Latvia) under permission No. 01-29.1/3 (dated, 10.07.2013), Nr.A-17/19-10-17 (dated, 17.10.2019), Nr. 01-29.1.2/3274 (dated 23.04.2021). Biobanking and sample storage followed the protocols of the Genome Database of the Latvian population [26]. The study complies with the principles of the 1964 Declaration of Helsinki and its amendments. Written informed consent was obtained from all participants prior to inclusion. Recruitment for this study occurred from October 1, 2021, to July 30, 2022, at the University of Latvia in Riga (Pilsonu 13 Str., Building 10). Information about the study was shared through the University of Latvia’s website and social media channels.

Additional exclusion criteria for a substudy of LatDiane on CGM reported here included pregnancy, a history of inflammatory bowel disease (e.g., Crohn’s disease or ulcerative colitis), celiac disease (detected through serum transglutaminase IgA screening), recent acute intestinal infection (within 2 months), clinical signs of acute inflammation, and fever.

On the study day, participants underwent anthropometric measurements, CGM sensor installation, and blood and urine sample collection. Data on diabetes history and co-morbidities was assessed based on medical files. Participants were fitted with blinded diagnostic FreeStyle Libre Pro iQ sensors (Abbott GmbH) and wore it continuously for 14 days. The CGM data analysis included standard CGM metrics (average glucose, coefficient of variation (CV), estimated HbA1c, glucose management indicator (GMI), percentage of time above range (TAR), percentage of time in range (TIR), percentage of time below range (TBR), and the frequency of low glucose events). TIR was defined as the proportion of time (%) that glucose levels remained between 3.9 and 10 mmol/L, in line with standard recommendations for individuals with T1D [4].

Blood pressure (BP) was measured for all participants, with arterial hypertension defined as systolic BP ≥ 140 mmHg (18.7 kPa), diastolic BP ≥ 90 mmHg (12.0 kPa), or antihypertensive medication use. Smoking status was self-reported, with smokers defined as those who smoke ≥ 1 cigarette daily. Cardiovascular disease (CVD) and diabetes-related complications (retinopathy, neuropathy, diabetic kidney disease (DKD)) were identified through medical records. CVD included myocardial infarction, revascularization, stroke, amputation, or peripheral vascular disease. Albuminuria was assessed by the albumin-to-creatinine ratio from two of three morning urine samples. eGFR was calculated using the CKD-EPI equation [27]. End-stage renal disease (ESRD) was defined as eGFR < 15 mL/min/1.73 m², dialysis or kidney transplantation.

Metabolic syndrome (MS**)** was defined according to the International Diabetes Federation (IDF) consensus criteria [28]. For our European cohort, the central obesity waist circumference thresholds were set at ≥ 94 cm for men and ≥ 80 cm for women. Serum triglyceride levels ≥ 1.7 mmol/L, serum high-density lipoprotein (HDL) levels < 1.0 mmol/L for men and < 1.3 mmol/L for women, or the use of medications to manage dyslipidemia, satisfied the lipid-related criteria. Blood pressure exceeding 130/85 mmHg or the use of antihypertensive medications met the blood pressure criterion. For individuals with T1D, the elevated fasting blood glucose criterion was universally met. MS was diagnosed when at least three of these criteria were present.

Estimated glucose disposal rate (eGDR) allows for estimation of insulin resistance in patients with T1D. The lower eGDR, the higher the insulin resistance.

$$ \begin{aligned} {\mathrm{GDR}} = & 24.4 - 12.97 \times \:{\mathrm{Waist}}/{\mathrm{Hips}} \\ & - 3.39 \times \:{\mathrm{Hypertension}} \\ & - 0.6 \times \:{\mathrm{HbA}}1{\mathrm{c}}\% , \\ \end{aligned} $$

If blood pressure was equal to or greater than 140/90 mmHg and/or the participant regularly used antihypertensive medication, hypertension was coded as 1; otherwise, it was coded as 0 [29, 30].

Surrogate hepatic steatosis indices, including the hepatic steatosis index (HSI) and fatty liver index (FLI), were calculated using established formulas.

$$ \begin{aligned} H{\mathrm{SI}} = & 8 \times \:{\mathrm{ALT}}/{\mathrm{AST}} \\ & + {\mathrm{BMI}} + 2\:\left( {{\mathrm{if}}\:{\mathrm{DM}}} \right) \\ & + 2\left( {{\mathrm{if}}\:{\mathrm{female}}} \right), \\ \end{aligned} $$

with values < 30 ruling out and values ≥ 36 ruling in steatosis [13], and

$$ {\mathrm{FLI}} = {\mathrm{logistic}}\left( \begin{gathered} 0.953 \times \:ln\left( {{\mathrm{TG}}} \right) + 0.139 \times \:{\mathrm{BMI}} + 0.718 \hfill \\ + ln\left( {{\mathrm{GGT}}} \right) + 0.053 \times \:{\mathrm{Waist}} - 15.745 \hfill \\ \end{gathered} \right) \times \:100, $$

where *logistic(x) = 1/(1 + exp(-x))* denotes the logistic function and *ln(x)* the natural logarithm. Values < 30 rule out, and values ≥ 60 rule in steatosis [31].

### Blood samples and inflammatory markers

Blood and morning urine samples were sent to a certified lab for analysis of clinical markers, including C-reactive protein (CRP). Peripheral venous blood was drawn for serum preparation. After 30 min of incubation at room temperature, samples were centrifuged, and the serum was separated, transferred to 2 mL tubes, frozen, and stored at − 20 °C for later analysis of intestinal permeability, endotoxaemia and inflammatory markers lipopolysaccharide (LPS), lipopolysaccharide-binding protein (LBP), anti-endotoxin core antibodies IgG and IgM (EndoCAb IgG and EndoCAb IgM), procalcitonin [32].

In serum, the LPS activity was measured using a Hycult LAL chromogenic endpoint assay (HIT302, Hycult Biotech, Uden, The Netherlands). EndoCAb IgG and EndoCAb IgM were measured using Hycult EndoCAb IgG and IgM Elisa kits (HK504-IGG; HK504-IGM; Hycult Biotech, Uden, The Netherlands). LPB was measured using a Hycult LPB Human Elisa kit (HK315-02, Hycult Biotech, Uden, The Netherlands). Procalcitonin was measured by CLIA Kit (MBS2530785, Mybiosourse, San Diego, USA). Measurements were performed according to the manufacturer’s instructions [32].

### Data processing and statistical analysis

No formal a priori sample size calculation was performed because this was an exploratory sub-study based on participants with available CGM and biomarker data. All data processing and analysis were carried out using the statistical software R version 4.4.0 (last accessed on January 4, 2025) and IBM SPSS version 22 (last accessed on January 6, 2025).

Endotoxaemia and inflammatory marker (LPS, LBP, EndoCAb IgG, EndoCAb IgM, procalcitonin) measurements were carried out on three plates. A comparison between these plates revealed that the LBP, EndoCAb IgG, and EndoCAb IgM measurements on one plate were significantly different from those on the other two plates (Kruskal-Wallis test p-values < 0.001). Consequently, a normalization procedure based on a location-scale transformation was applied before further analysis, as suggested in [33] and previously utilized for the same purpose in [19].

The six least correlated CGM summary metrics—average glucose levels, CV, %TAR, %TIR, %TBR, and the frequency of low glucose events—were used to cluster 75 T1D patients into groups with distinct glucose level control patterns. Prior to clustering, the data were scaled, and principal components were calculated. Hierarchical agglomerative clustering using Ward’s minimum variance linkage method and Euclidean distance was applied. The optimal number of clusters was two and it was determined using 30 indices provided by the R library *NbClust* and further validated using the traditional elbow method. We named the two resulting clusters based on their clinical characteristics: (1) the group with poorly controlled diabetes and (2) the group with moderately controlled diabetes.

The normality of continuous variables was assessed using the Shapiro-Wilk test. Most variables violated the normality assumption; therefore, medians with interquartile ranges (IQR; 1st to 3rd quartile) are reported. The Mann-Whitney U (Wilcoxon rank-sum) test was used for two-sample comparisons, and Spearman’s rank correlation coefficient was employed in the correlation analysis. Two-group comparisons for the main variables of interest (liver indices and inflammatory markers) were performed using a rank-based ANCOVA approach with adjustment for age and sex. Categorical variables are reported as counts and percentages (*N* (%)), and the chi-square proportion test was used to assess the equality of two proportions.

The associations between diabetes control status (poorly or moderately controlled diabetes), endotoxaemia and inflammatory markers, and MASLD indices (FLI and HSI) were explored using a causal mediation analysis approach consisting of two linear regression models. Diabetes control status was considered the exposure variable, MASLD indices were considered mediators, endotoxaemia and inflammatory markers (LPS, CRP, LBP, EndoCAb IgG, EndoCAb IgM, and procalcitonin) were considered outcome variables in these models, resulting in 12 separate cases of mediation. Sensitivity analysis was conducted using nonparametric bootstrap simulations with a fixed random seed and 5,000 replications, implemented via the R library *mediation*. All continuous variables were standardized prior to the analysis. Additional sensitivity analyses for the mediation models were conducted by adjusting both the mediator and outcome models for age and sex as covariates.

P-values below the 5% level are reported as significant, while those between 5% and 10% are reported as marginally significant.

## Results

### Characteristics of the overall patient cohort

The sociodemographic, clinical, and continuous glucose monitoring (CGM) characteristics of the cohort (*n* = 75) are summarized in Supplemental Tables 1 and 2. Briefly, the majority of the subjects were women, comprising 44 individuals (58.7%). The median age of the participants was 43.5 years (34.0-52.8 years), smoking was reported by 20 individuals (26.7%). The median body mass index (BMI) was 24.5 kg/m², the median duration of diabetes in the cohort was 23.5 years, and the median HbA1c was 8.1%. Diabetes-related complications were prevalent, with AH reported in 44 patients (58.7%), retinopathy in 39 patients (52.0%), CVD in 14 patients (18.7%). The majority of participants were normoalbuminuric (53.3%), while microalbuminuria, macroalbuminuria/ESRD were present in 28.0% and 18.7% respectively. Six patients (8.0%) had undergone kidney transplantation, and eight patients (10.7%) were on dialysis. CGM parameters (Suppl. Table 2) revealed a median average glucose level of 9.6 mmol/L (8.1–11.4), and GMI of 7.5% (6.8–8.2). TIR was 50.0% (37.0–65.3), with TAR at 43.0% (28.0–59.0) and TBR at 4.0% (2.0–9.5). Low glucose events were reported at a median of 9 (4–15) with a median duration of 109 min (76–132). The estimated A1c was 7.7% (6.7–8.7).

### Clustering results and characteristics of resulting study groups

**Fig. 1:**
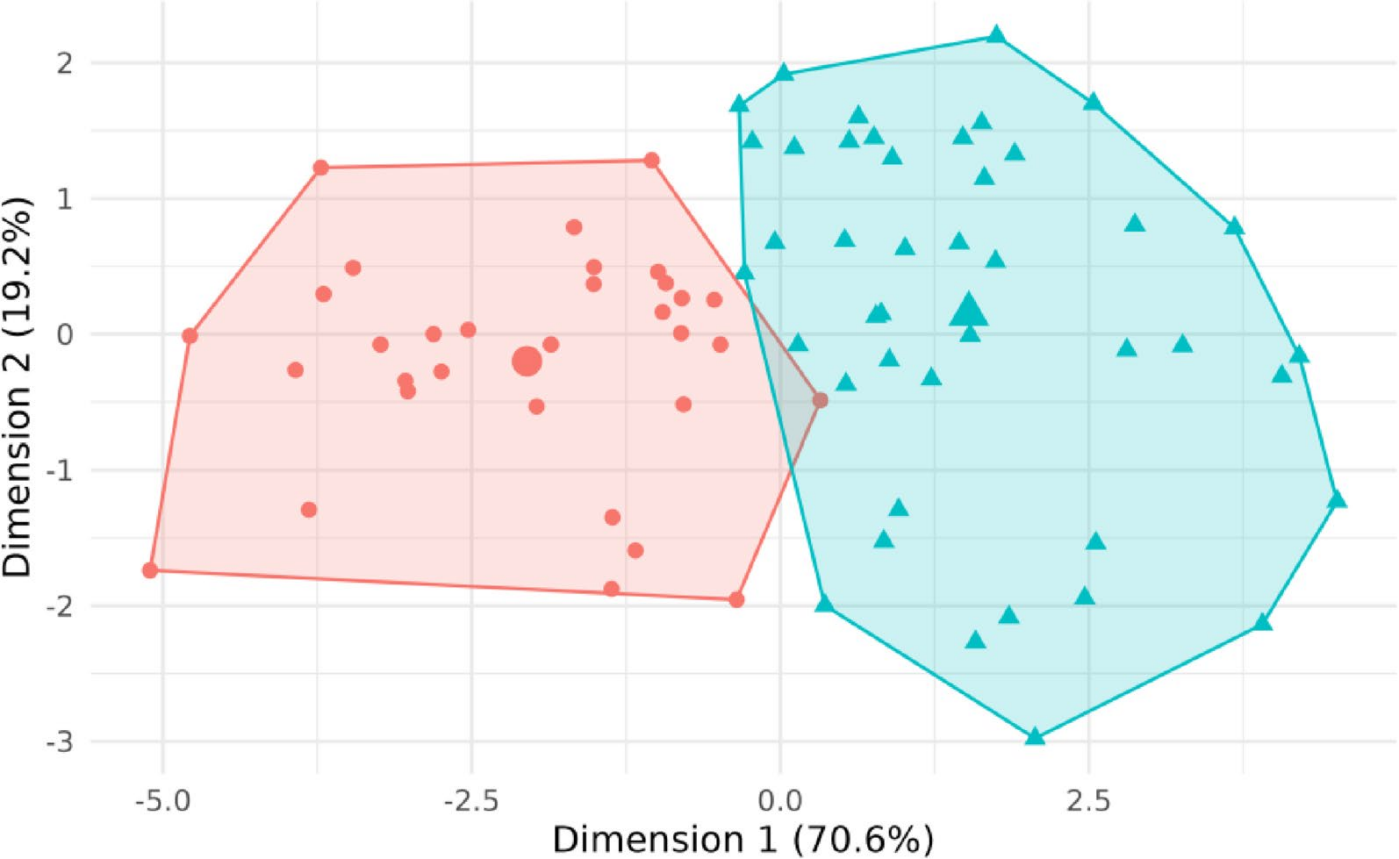
A visualization of 75 subjects with type 1 diabetes clustered based on CGM metrics using hierarchical agglomerative clustering with Ward’s minimum variance linkage method and a Euclidean distance matrix. The points in the scatterplot display two obtained clusters of patients distributed across two dimensions (with variance explained indicated in parentheses), where red circles and blue triangles represent subjects with *poorly* (*n* = 28) and *moderately* (*n* = 47) *controlled diabetes*, respectively

**Fig. 2:**
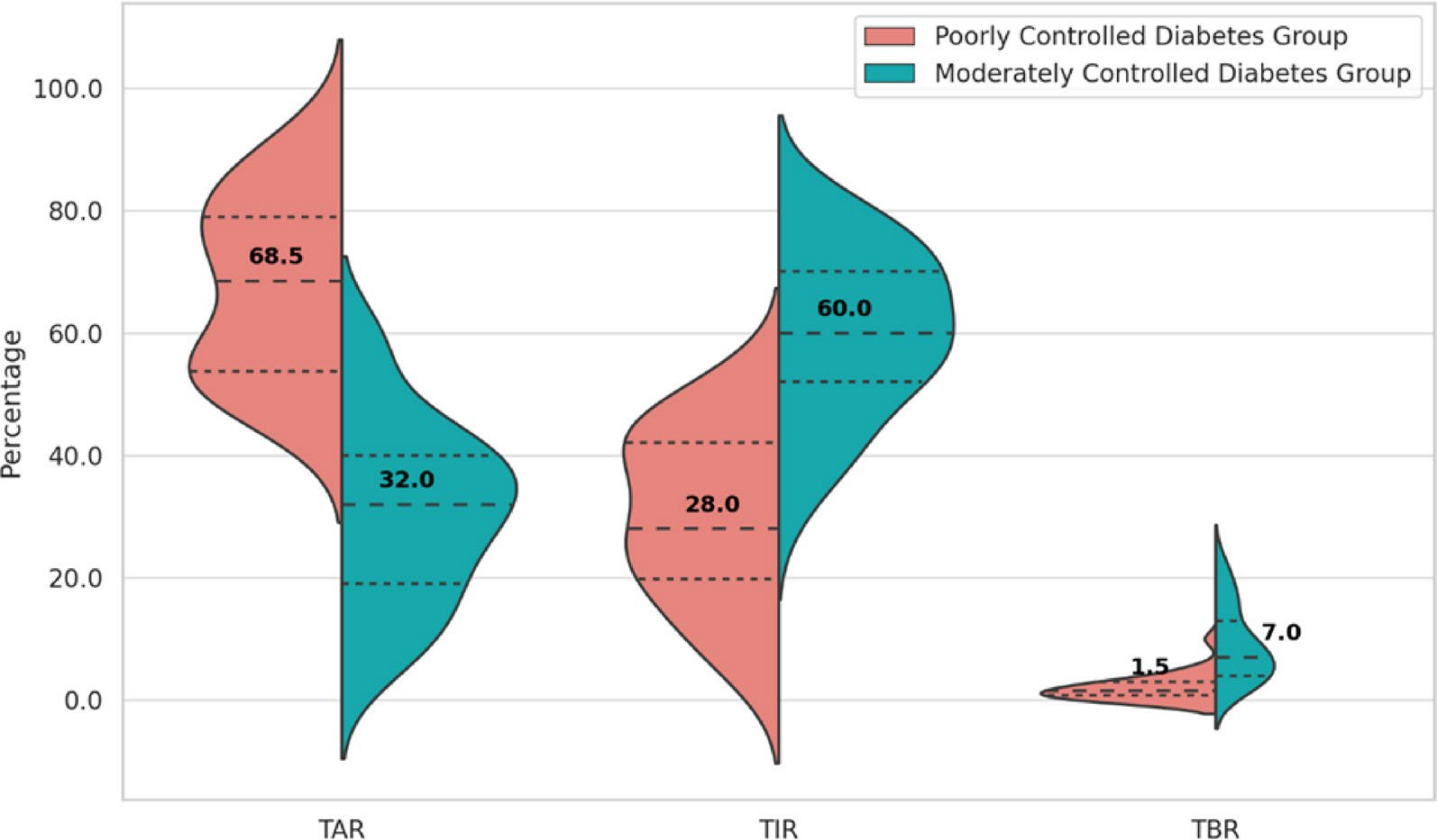
CGM metrics by study groups. Each segment of the violin plot represents the distribution of the percentage of time spent in each glucose range, with the median indicated for participants within each respective study group. TAR – time above range. TIR – time in range, TBR – time below range

Clustering of CGM parameters resulted in two distinct clusters of participants (see Fig. 1 for a two-dimensional representation of the clusters, Supplemental Fig. 1 for a radar chart depicting the average percentage levels of seven CGM metrics, and Supplemental Fig. 2 for density plots of CGM metrics by cluster). Based on CGM characteristics (Fig. 2) and HbA1c levels in both clusters (Table 1), we designated the resulting groups as (1) the poorly controlled diabetes group (PCD, *n* = 28) and (2) the moderately controlled diabetes group (MCD, *n* = 47).

Participants in the PCD group exhibited significantly higher mean glucose levels (*p* < 0.001), GMI (*p* < 0.001), and TAR (*p* < 0.001) compared to individuals in the MCD group. Conversely, TIR was shorter (*p* < 0.001) and TBR was less frequent (*p* < 0.001) in the PCD group compared to the MCD group. Also, the PCD group had fewer low glucose events (*p* < 0.001), which were of shorter duration (*p* = 0.004) compared to the MCD group. Estimated HbA1c was also significantly higher in the poorly controlled diabetes group (*p* < 0.001) (Table 1; Fig. 2).

PCD and MCD groups showed no significant differences in median age and sex distribution, diabetes duration, prevalence of diabetes complications and hypertension, serum cholesterol levels (Table 1). However, the PCD group included fewer normoalbuminuric participants (42.9% vs. 59.6%), suggesting a trend toward more advanced kidney involvement.

**Table 1:** Comparison of anthropometric and clinical characteristics by diabetes control groups identified by clustering

Disease-related indicators		Poorly controlled diabetes (*n* = 28)	Moderately controlled diabetes (*n* = 47)	*P*-value
Male, *N* (%) *		10 (35.7%)	21 (44.7%)	0.45
Age, years,		46 (36–54)	43 (33–51)	0.22
Body mass index, kg/m^2^		27.4 (23.6–29.1)	24.1 (22.7 − 26.6)	0.079
Waist-to-height ratio		0.54 (0.46–0.58)	0.48 (0.45–0.55)	0.050
Smoking, *N* (%) *		16 (34.0%)	16 (34.0%)	0.17
Length of diabetes, Years,		24.5 (19.5–30.5)	25.0 (13.0–34.5)	0.71
Cardiovascular disease, *N* (%) *		7 (14.9%)	7 (14.9%)	0.28
Hypertension, *N* (%) *		20 (71.4%)	24 (51.1%)	0.083
Retinopathy, *N* (%) *		14 (50.0%)	25 (53.2%)	0.79
Albuminuria classification	Normoalbuminuric	12 (42.9%)	28 (59.6%)	0.13
	Microalbuminuria	11 (39.3%)	10 (21.3%)	
	Macroalbuminuria/ESRD	5 (17.9%)	9 (19.2%)	
Albuminuria, mg/mmol		3.85 (0.66–20.86)	1.33 (0.62–5.56)	0.14
Metabolic syndrome		19 (67.9%)	20 (42.6%)	0.034
Hemoglobin A1c, %		9.25 (8.73–10.45)	7.60 (7.10 − 8.50)	< 0.001
Estimated glucose disposal rate, mg/kg/min		4.4 (3.4–7.3)	7.3 (4.7–9.8)	0.001
Short-acting insulin units/kg		0.33 (0.25–0.47)	0.37 (0.28–0.45)	0.465
Long-acting insulin units/kg		0.33 (0.27–0.43)	0.27 (0.23–0.33)	0.011
Total insulin units/kg		0.67 (0.60–0.93)	0.63 (0.56–0.73)	0.083
High-density lipoprotein cholesterol, mmol/l		1.78 (1.41–2.06)	1.76 (1.60–2.17)	0.50
Low-density lipoprotein cholesterol, mmol/l		2.62 (1.83–3.82)	2.70 (2.10–3.38)	0.87
Total cholesterol, mmol/l		4.97 (4.11–6.40)	4.93 (4.17 − 5.48)	0.55
Triglycerides, mmol/l		1.35 (0.76–2.29)	0.93 (0.71 − 1.35)	0.052
eGFR ml/min/1.73m^2^		93 (61–111)	100 (73 − 111)	0.61
**CGM Parameters**				
Average glucose levels, mmol/l		12.6 (10.7–13.9)	8.7 (7.6–9.4)	< 0.001
Coefficient of variation (CV)		37.5 (34.4–40.5)	41.6 (36.6–49.4)	0.002
Glucose management indicator (GMI) %		8.8 (7.9–9.3)	7.0 (6.6–7.4)	< 0.001
% Time above range (TAR)		68.5 (53.75–79)	32 (19–40)	< 0.001
% Time in range (TIR)		28 (20–42)	60 (52–70)	< 0.001
% Time below range (TBR)		1.5 (0.8–3.0)	7.0 (4.0–13.0)	< 0.001
Low glucose events		4 (2–6)	14 (9–19)	< 0.001
Low glucose events average duration in min		85 (49–113)	115 (85 − 155)	0.004
Estimated A1c%		9.55 (8.35–10.40)	7.10 (6.35 − 7.55)	< 0.001

Continuous variables are presented as medians (*q25* - *q75*) and were evaluated using the Mann-Whitney U test. ; LPS – lipopolysaccharides; ESRD – end-stage renal disease; eGFR – estimated glomerular filtration rate; EndoCAb IgG/IgM – endotoxin core antibody immunoglobulin G/M; LPS – lipopolysaccharide Categorical variables marked with * are presented N (%) and were compared using the chi-square test for proportions

**Table 2:** Inflammation, intestinal permeability and liver related markers in study groups

Related indicators	Poorly controlled diabetes (*n* = 28)	Moderately controlled diabetes (*n* = 47)	*p*-value
C- reactive protein, mg/l	1.82 (0.88–3.10)	0.81 (0.52–1.71)	0.041
LPS activity, EU/ml	0.611 (0.543 − 0.720)	0.540 (0.500 − 0.670)	0.18
Procalcitonin, ng/ml	2.85 (0.61–3.60)	2.70 (0.37 − 4.33)	0.656
Lipopolysaccharide-binding protein, EU/ml	11.7 (8.9–16.8)	8.4 (6.3 − 12.7)	0.031
EndoCAb Immunoglobulin G, GMU/ml	66 (42–115)	75 (42–122)	0.683
EndoCAb Immunoglobulin M, MMU/ml	33 (20–43)	30 (22–44)	0.925
LPS to HDL ratio	0.360 (0.248 − 0.483)	0.327 (0.260 − 0.424)	0.331
Alanine transaminase, U/L	26 (23–38)	20 (16–27)	0.003
Asportate aminotransferase, U/L	28 (23–37)	23 (19–32)	0.049
Gamma-glutamyl transferase, U/L	20 (14–43)	15 (12–20)	0.075
Hepatic steatosis index	37 (35–41)	35 (33–38)	0.056
Fatty liver index	29 (11–76)	17 (8–30)	0.036
Hepatic steatosis index ≥ 36, *N* (%) *	18 (64.286%)	19 (40.426%)	0.046
Fatty liver index ≥ 60, *N* (%) *	10 (35.714%)	5 (10.638%)	0.009

Continuous variables are presented as medians (*q25* - *q75*) and were evaluated using ANCOVA on ranks adjusted for age and sex. Categorical variables marked with * are presented as *N* (%) and were compared using the chi-square test for proportions

### Insulin resistance and inflammatory signatures in the study groups

As indicated in Table 1, the PCD group had a numerically higher median BMI (27.4 (23.6–29.1) kg/m² vs. 24.1 (22.7–26.6) kg/m², *p* = 0.079), a higher waist-to-height ratio (0.54 (0.46–0.58) vs. 0.48 (0.45–0.55), *p* = 0.050). In addition, MS was significantly more prevalent in the PCD group (67.9% vs. 42.6%, *p* = 0.034) and insulin sensitivity was lower in PCD group, as indicated by lower eGDR (*p* = 0.001). In addition, PCD group demonstrated greater basal insulin daily doses per kg weight (*p* = 0.011) and higher levels of serum triglycerides (*p* = 0.052), along with higher HbA1c (*p* < 0.001), which was consistent with lower insulin sensitivity in the PCD group. Finally, PCD group also exhibited higher CRP (*p* = 0.041) and LBP (*p* = 0.031) levels, indicating higher level of systemic inflammation and endotoxaemia (see Table 2).

### Liver markers and MASLD risk stratification in the study groups

The PCD group had higher liver enzyme levels than the MCD group (*p* = 0.003 for ALT; *p* = 0.049 for AST) and higher hepatic steatosis indices (*p* = 0.056 for HSI; *p* = 0.036 for FLI) (Table 2, To further evaluate the distribution of hepatic steatosis risk across glycemic control groups, participants were categorized by FLI and HSI risk. In the PCD group, a significantly greater proportion of individuals fell into the high-risk category for hepatic steatosis (FLI ≥ 60) compared to the MCD group (35.7% vs. 10.6%, *p* = 0.009). A similar result was observed with the HSI index, where a greater proportion of participants in the PCD group exceeded the threshold indicating hepatic steatosis (HSI ≥ 36) than in the MCD group (64.3% vs. 40.4%, *p* = 0.046) (Table 2).

### Correlations between CGM parameters, liver markers, and endotoxaemia in the whole cohort

**Fig. 3:**
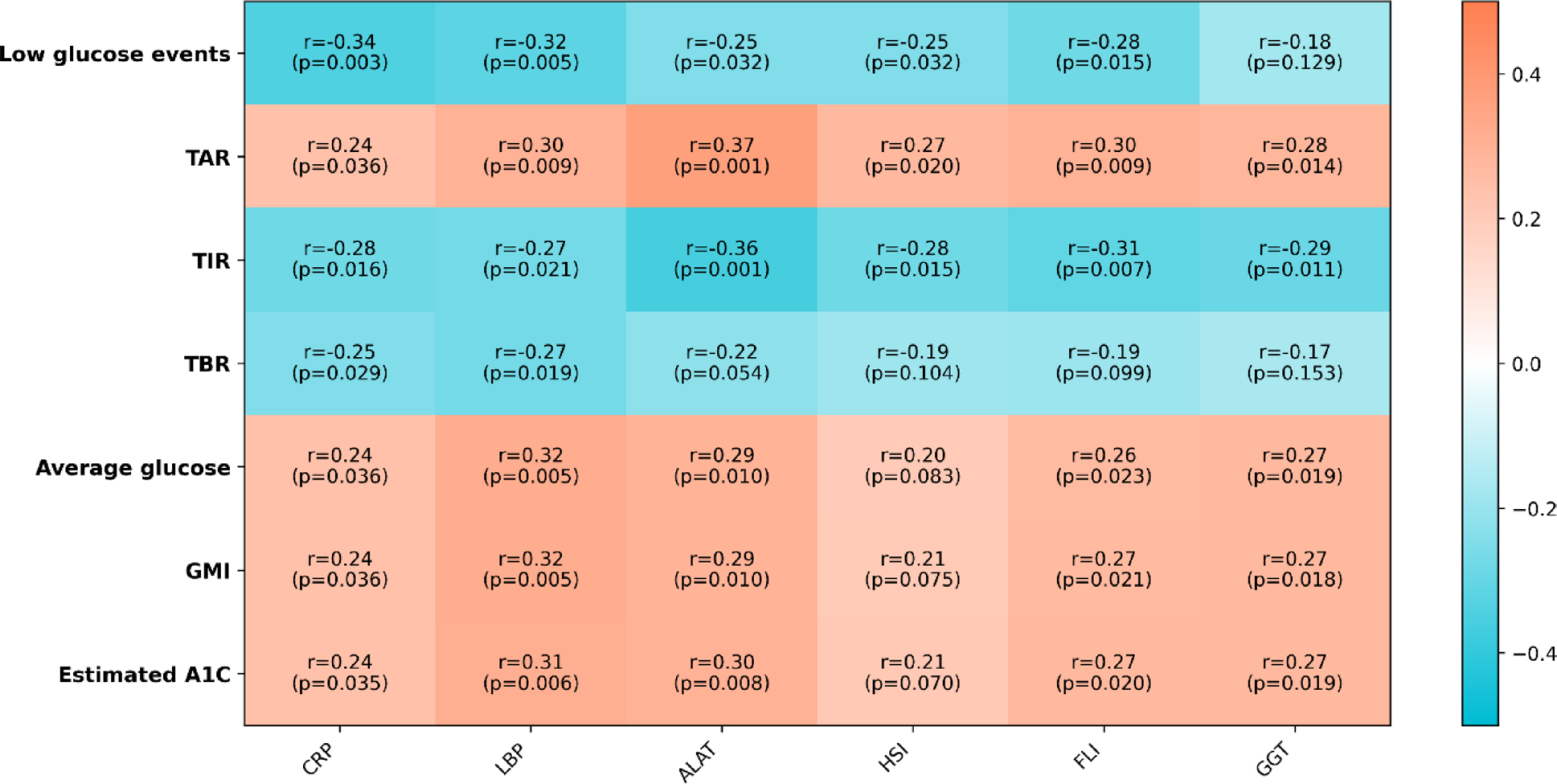
Spearman correlations between CGM metrics, liver markers and systemic inflammation in the whole cohort

We analyzed correlations between CGM metrics, liver markers, and systemic inflammation in the whole cohort to investigate associations between daily glycaemic profiles, MASLD risk, and endotoxaemia (Fig. 3 Supplementary Table 3).

Significant positive correlations were observed between TAR and ALT, GGT, FLI, HSI, CRP, and LBP, whereas TIR correlated negatively with the same variables. The frequency of low glucose events correlated negatively with FLI, HSI, CRP, and LBP. Mean glucose, GMI, and estimated A1c showed positive correlations with liver enzymes (ALT, GGT), FLI, and LBP.

### Study group-specific correlations between liver markers, endotoxaemia and inflammatory markers

**Fig. 4:**
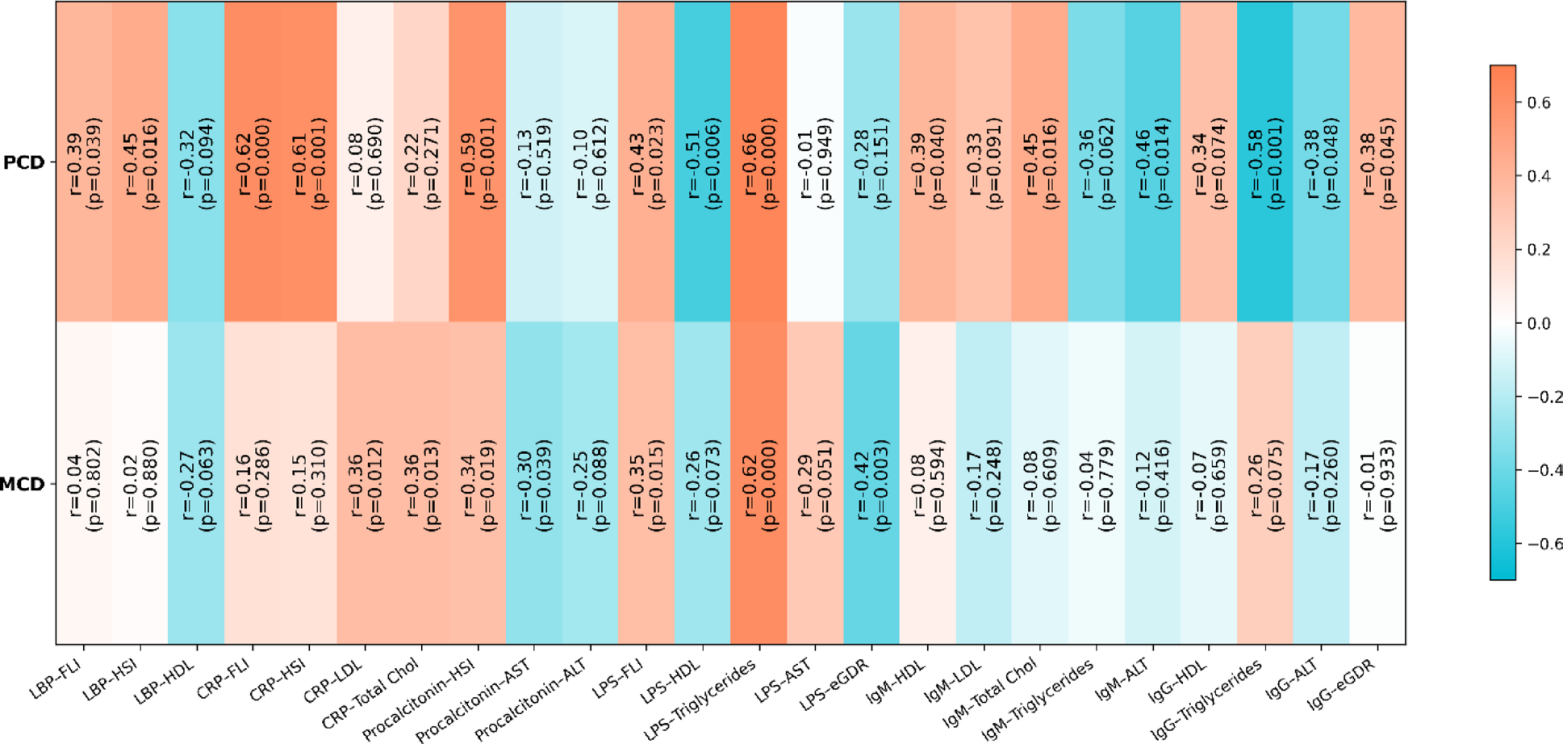
Study group–specific Spearman correlations between liver markers, endotoxemia, and inflammatory markers. *IgG* represents EndoCAb IgG, and *IgM* represents EndoCAb IgM

We next analyzed relationships between endotoxaemia, inflammation, liver, and insulin resistance markers in the PCD and MCD groups separately to explore whether these associations differed by CGM-derived phenotype (Fig. 4 Supplementary Table 4.).

In the PCD group, moderate positive correlations were observed between LBP and hepatic steatosis indices (FLI, HSI), and between CRP and both indices. EndoCAb IgM correlated positively with HDL, total cholesterol, and ALT, while EndoCAb IgG correlated negatively with triglycerides and ALT.

In contrast, in the MCD group, significant positive correlations were found between CRP and LDL and total cholesterol. In addition negative correlations were found between procalcitonin and AST, and LPS and eGDR in MCD group.

Across both groups, consistent positive associations were observed between procalcitonin and HSI, LPS and FLI, and LPS and triglycerides.

### Exploratory mediation analysis

Further, we used mediation analysis to explore whether poor glycaemic control contributes to inflammation and MASLD risk.

**Fig. 5:**
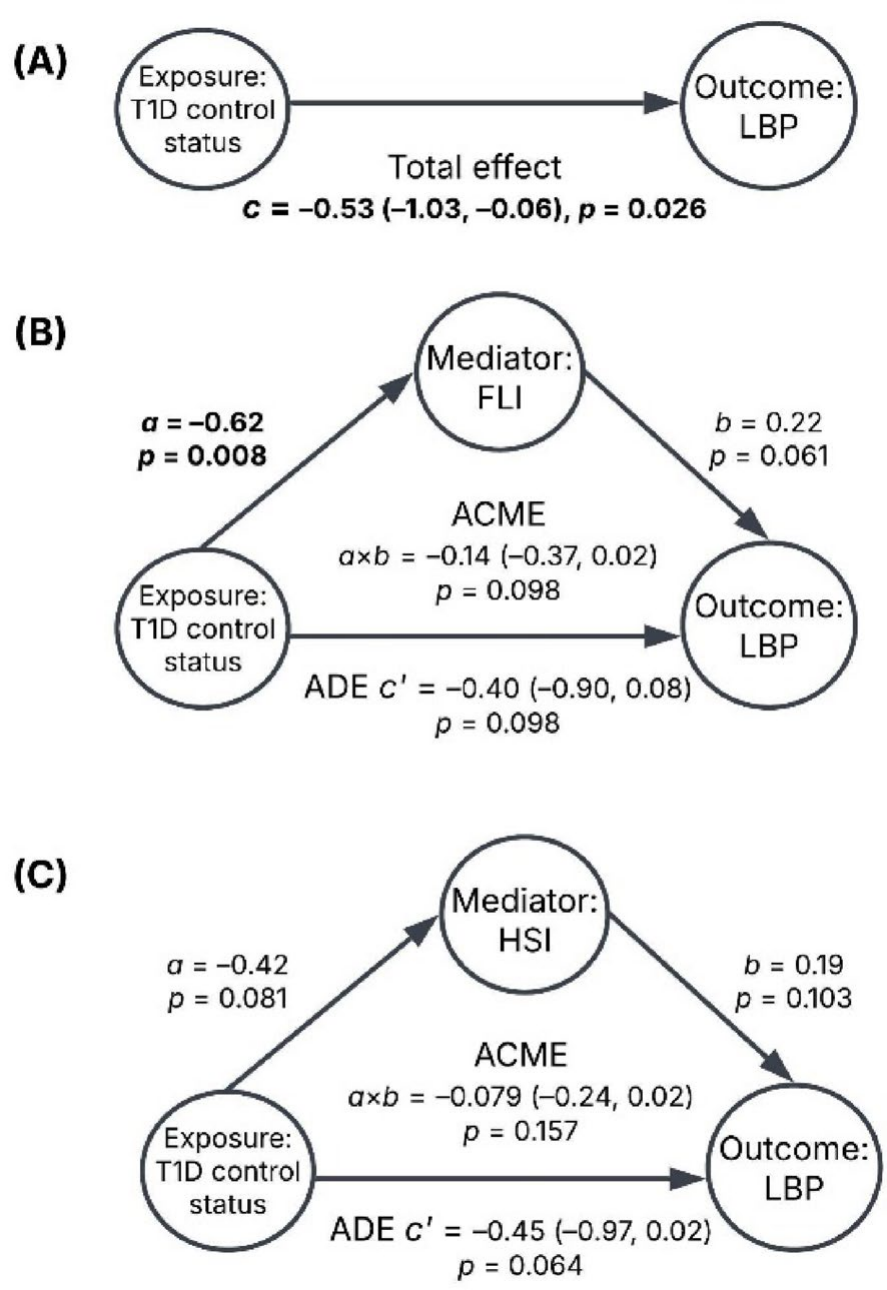
Diagrams demonstrating the results of exploratory mediation analysis for models with T1D control status as the exposure, fatty liver index (FLI) and hepatic steatosis index (HSI) as mediators, and lipopolysaccharide binding protein (LBP) as the outcome variable. **(A)** Total effect of the exposure on the outcome; **(B)** Average direct effect (ADE) of the exposure on the outcome and average causal mediation effect (ACME) or indirect effect of the exposure on the outcome, adjusted for the mediator FLI; **(C)** Average direct effect (ADE) of the exposure on the outcome and average causal mediation effect (ACME) or indirect effect of the exposure on the outcome, adjusted for the mediator HSI. Sensitivity analysis was conducted using the R package *mediation*, based on 5,000 bootstrap resamples and a fixed random seed

Mediation analyses were performed for 12 models in total, with glycaemic control status (poor or moderately controlled) as the exposure variable, MASLD indices (FLI or HSI) as the mediators, and endotoxemia or inflammation markers (LBP, CRP, hsCRP, IgM, IgG, LAL, ProC) as outcome variables. Results for six models that showed nominally significant or borderline effects are summarized in Table 3. Three mediation diagrams describing the models with LBP as the outcome variable are shown in Fig. 5 (a) the total effect of the exposure on LBP, Fig. 5 (b) the mediation effect with FLI as the mediator, and Fig. 5 (c) the mediation effect with HSI as the mediator.

Borderline significant mediation effects were observed with FLI as a mediator between CGM-based glycaemic control and LBP (ACME: *p* = 0.098; total effect: *p* = 0.026). A significant indirect-only effect was found for CRP (ACME: *p* = 0.029; total effect: *p* = 0.66). Similarly, LPS activity was influenced by glycaemic control through FLI (ACME: *p* = 0.023; total effect: *p* = 0.47). FLI also mediated effects of glycaemic control on EndoCAb IgM, despite the absence of a total effect (ACME: *p* = 0.036; total effect: *p* = 0.82).

When HSI was used as the mediator, its effects were weaker compared to FLI. HSI showed a borderline indirect effect on procalcitonin (ACME: *p* = 0.076; total effect: *p* = 0.77), while no mediation effect was found for LBP (ACME: *p* = 0.157; total effect: *p* = 0.026).

Additional sensitivity analyses were conducted with adjustment for age and sex in all models. The associations with LBP remained largely consistent with the unadjusted analyses for both FLI (ACME: *p* = 0.031; total effect: *p* = 0.028) and HSI (ADE: *p* = 0.058). Similarly, the indirect effect of FLI on CRP remained significant after adjustment (ACME: *p* = 0.020). In contrast, the mediation effects of FLI on EndoCAb IgM and LPS activity were no longer significant after adjustment for age and sex, as was the indirect effect of HSI on ProC. Full results are presented in Supplementary Table 6.

**Table 3:** Exploratory mediation analysis results

Mediation model	Mediator	Outcome	ACME (a×b)	ADE (c’)	Total effect (c’+a×b)
I	FLI *a*=-0.62, *p* = 0.008	LBP *b* = 0.22, *p* = 0.061	-0.14 (-0.37, 0.02), *p* = 0.098	-0.40 (-0.90, 0.08), *p* = 0.098	-0.53 (-1.03, -0.06), *p* = 0.026
II		CRP *b* = 0.32, *p* = 0.009	-0.20 (-0.54, -0.01), *p* = 0.029	0.117 (-0.41, 0.76), *p* = 0.79	-0.08 (-0.47, 0.36), *p* = 0.66
III		Endo Cab IgM *b*=-0.24, *p* = 0.053	0.15 (0.01, 0.38), *p* = 0.036	-0.11 (-0.63, 0.38), *p* = 0.70	0.04 (-0.46, 0.51), *p* = 0.82
IV		LPS *b* = 0.22, *p* = 0.071	-0.14 (-0.36, -0.01), *p* = 0.023	-0.005 (-0.38, 0.44), *p* = 0.96	-0.14 (-0.54, 0.29), *p* = 0.47
V	HSI *a* =-0.42, *p* = 0.081	LBP *b* = 0.19, *p* = 0.103	-0.079 (-0.24, 0.02), *p* = 0.16	-0.45 (-0.97, 0.02), *p* = 0.064	-0.53 (-1.03, -0.06), *p* = 0.026
VI		ProC *b* = 0.34, *p* = 0.004	-0.14 (-0.42, 0.01), *p* = 0.076	0.21 (-0.22, 0.67), *p* = 0.33	0.06 (-0.39, 0.50), *p* = 0.77

Mediation analysis models for exploratory purposes were constructed using T1D control status as the exposure variable, MASLD indices as mediators, and endotoxaemia and systemic inflammation markers as outcome variables (see also mediation diagrams for Models I and V in Fig. 5 as examples). Results are presented as estimates of path coefficients *a* and *b* from linear regression models, average causal mediation effects (ACME), average direct effects (ADE) and total effects along with corresponding 95% CI. Only six models out of 12 are presented, as they exhibited significant effects. Sensitivity analysis was carried out using the R package *mediation* with 5,000 bootstrap resamples and a fixed random seed. FLI – fatty liver index, HSI – hepatic steatosis index, LBP – lipopolysaccharide-binding protein, LPS – lipopolysaccharides, CRP – C-reactive protein, EndoCAb IgM – immunoglobulin M, ProC – procalcitonin

## Discussion

The findings of this study suggest that CGM-derived phenotyping may provide information beyond conventional assessment of glycaemic control alone and may help characterise individuals with less favourable metabolic profiles.

Clustering procedure identified distinct groups of subjects, who were similar in age, sex distribution and duration of diabetes, but significantly differed not only in CGM metrics, but also in variables associated with liver health, endotoxaemia, inflammation and insulin resistance. Although participants in both PCD and MCD groups had not well controlled diabetes, individuals in PCD group had poorer CGM metrics and higher HbA1c, as compared to MCD group, with TIR of 28% and HbA1c above 9%, while TIR in MCD group was 60% and HbA1c – 7.6. Previous studies have also used clustering for CGM data analysis [5]. For example, Fagherazzi et al. identified seven distinct glycaemic CGM profiles in 618 T1D subjects associated with socioeconomic factors, cardiovascular risk indicators, retinopathy and treatment strategy of diabetes [7]. The differences in the number of identified subject groups can be explained by differences in clustering methodology and higher participant number in the above study (618 versus 75 in our study) [24]. In the present cross-sectional study, CGM-derived phenotypes were associated with low-grade inflammation, surrogate markers of MASLD, and metabolic syndrome, in line with previous reports [21, 34, 35]. Together, these findings support the concept that CGM data may help identify individuals with less favourable metabolic and inflammatory profiles in T1D; however, prospective studies are required before any predictive utility can be assumed. One could argue that it is easy to diagnose MS in clinical practice, as it requires evaluating only five routinely measured parameters. Similarly, calculation of MASLD indices also requires several blood chemistry parameters, anthropometric measures and an online calculator [13]. However, in the context of T1D, CGM is becoming part of routine care, providing long-term real-time measurements with a possibility to share the data with medical professionals, without a necessity for blood withdrawal and onsite visit. Therefore, the ability to uncover concomitant conditions and pathophysiological subtypes of T1D from these data seems to be a promising direction for research and clinical applications in the future [5, 8].

The observation that CGM-based clustering identified groups with differing liver markers, surrogate-marker-defined MASLD risk, inflammatory markers, endotoxaemia-related markers, and insulin sensitivity is of clinical interest. In our cohort, poorer CGM-derived glycaemic control was associated with higher LBP and CRP levels and with less favourable metabolic characteristics, which is consistent with a link between dysglycaemia, inflammation, and metabolic dysfunction. We observed not only higher levels of CRP and LBP in PCD group, but also statistically significant negative correlations between TIR, LBP and CRP and positive ones - between TAR, CRP and LBP. Previously, a small study with 17 adolescents with T1D reported associations between levels of inflammation and CGM parameters [22], in line with our findings. However, as to our knowledge, few studies have investigated functional markers of intestinal permeability (e.g., endotoxaemia markers) in relation to CGM data before.

Despite a median BMI within the normal range (24.45 kg/m²) in our cohort, nearly half of participants (48%) met the diagnostic criteria for MS, a common condition in T1D in the recent decades, which is associated with poorer prognosis in relation to complications of diabetes [36]. Interestingly, clustering of the CGM data not only helped to identify the subjects with poor glycaemic control (PCD group) but also demonstrated that the prevalence of MS and high risk of MASLD as assessed by surrogate markers FLI and HSI, was higher in PCD group as compared to MCD group. Moreover, the PCD group demonstrated higher median BMI and waist-to-height ratio, indicators of central adiposity, and lower insulin sensitivity, as compared to MCD group, in agreement with previous findings [23, 34, 35]. Moreover, we observed stronger correlations between markers of systemic inflammation and endotoxaemia (CRP and LBP) with liver steatosis indices in the PCD group as compared to MCD group. These data support an association between poorer daily CGM profiles, MS, MASLD risk, and systemic inflammation [37], potentially involving oxidative stress [38], cytokine activation [39], and altered gut-barrier-related pathways [9, 13, 21, 37, 40–43].

We used exploratory mediation analysis to examine whether MASLD risk markers statistically accounted for associations between CGM-derived glycaemic control status, endotoxaemia, and inflammation. The obtained data suggest that hepatic steatosis risk assessed by FLI may partly account for the association between poorer glycaemic control and systemic inflammation in individuals with T1D. The analysis suggested that FLI statistically accounted for part of the association between diabetes control group and inflammatory and gut-permeability-related markers such as LBP and CRP. This result is consistent with recent studies demonstrating links between glycaemic control, glycaemic variability, inflammation, and MASLD [21, 44]. Notably, FLI showed stronger mediation effects than the HSI. Specifically, the estimated indirect effect for FLI on the relationship between glycaemic control and CRP was statistically significant (*p* = 0.029), while for HSI the corresponding mediation effect was not significant. Similarly, for LBP, FLI showed a borderline significant mediation (*p* = 0.098), whereas HSI mediation remained weaker and non-significant. These differences may be explained by the fact that FLI incorporates both triglyceride levels and anthropometric measures, such as BMI and waist circumference, which better reflect metabolic dysfunction and visceral adiposity. FLI also showed an indirect-effect pattern in the association between glycaemic control group and EndoCAb IgM, indicating potential impact on glycaemia on detoxification of LPS through MASLD related pathways [45]. The latter finding is consistent with a possible role of endotoxaemia-related pathways in MASLD pathogenesis [19, 44, 46]. Procalcitonin was demonstrated to be a predictor of non-viral hepatic disease in the general population [47, 48]. An elevated level of this marker was revealed in some diabetic ketoacidosis cases in T1D [49], even without underlying bacterial infection, which might indicate the link between procalcitonin level and glycaemia. In our study, HSI showed only a borderline indirect-effect pattern for procalcitonin, and this finding should therefore be interpreted cautiously.

This study has several limitations. First, its cross-sectional design does not permit causal inference; therefore, the observed differences should be interpreted as associations rather than evidence that poorer CGM profiles cause MASLD or inflammation. Longitudinal studies are required to confirm the temporal sequence between periods of unfavorable glycaemic control, development of MASLD, and inflammation. Second, the sample size was modest (*N* = 75), reflecting an exploratory sub-study with available CGM and biomarker data, and this limits statistical power, cluster stability, mediation analyses, and external validity. Third, residual confounding by adiposity, diet, physical activity, socioeconomic status, treatment patterns, intestinal microbiota composition, and other factors cannot be excluded. Fourth, detailed autoimmune antibody status, and data allowing separation of classical T1D from LADA-like phenotypes were not available for all participants. Finally, ethnic diversity was not assessed in the present analysis, and the findings should be validated in larger, ethnically diverse, and unselected cohorts. While exploratory mediation analysis provides useful insight into possible statistical pathways linking glycaemic control, hepatic steatosis risk markers, and systemic inflammation, the observed effect sizes were modest and some findings reached only borderline statistical significance. These results should be interpreted with caution and confirmed in prospective and interventional studies.

## Conclusion

In this exploratory cross-sectional cohort, clustering of CGM data identified a metabolic-inflammatory phenotype in T1D characterized by poorer glycaemic control, higher systemic inflammation and intestinal-permeability markers, lower insulin sensitivity, higher MS prevalence, and higher MASLD risk based on surrogate markers. From a clinical perspective, CGM-based phenotyping may help identify individuals who warrant closer metabolic risk assessment, but these findings require confirmation in larger and longitudinal cohorts before routine clinical implementation.

## Electronic Supplementary Material

Below is the link to the electronic supplementary material.

Supplementary material 1.

## Data Availability

Due to privacy restrictions, the data is unavailable publicly but is available from the corresponding author on request.
