# Supplementary material for: Continuous glucose monitoring metrics based clustering in people living with type 1 diabetes identifies phenotypes associated with higher inflammation, metabolic dysfunction-associated steatotic liver disease risk, and lower insulin sensitivity
Source: Diabetol Metab Syndr. 2026 May 25;18:159. doi: 10.1186/s13098-026-02191-3 (PMC13377698; doi:10.1186/s13098-026-02191-3)
Supplement: Supplementary file 1 — Supplementary material 1. [file 13098_2026_2191_MOESM1_ESM.docx]

**Supplements**

**Supplementary Table 1.** Anthropometric and clinical characteristics in the whole cohort.

| Disease-related indicators | | N =75 |
| --- | --- | --- |
| Male, N (%) | | 31 (41.3%) |
| Age, years, | | 43.5 (34 - 52.75) |
| Smoking, N (%) | | 20 (26.7%) |
| Body mass index, kg/m^2^ | | 24.45 (22.925 - 28.275) |
| Length of diabetes, Years, | | 23.5 (15.25 - 30.75) |
| Waist height ratio | | 0.49 (0.457-0.562) |
| High-density lipoprotein cholesterol, mmol/l | | 1.79 (1.465 - 2.145) |
| Low-density lipoprotein cholesterol, mmol/l | | 2.7 (2.113 - 3.39) |
| Total cholesterol, mmol/l | | 4.98 (4.228 - 5.537) |
| Triglycerides, mmol/l, | | 1.015 (0.715 - 1.397) |
| *Insulin Sensitivity and Metabolic Syndrome* | | |
| Estimated glucose disposal rate mg/kg/min | | 3.45 (1.025 - 5.5) |
| Short-acting insulin units | | 0.36 (0.28-0.46) |
| Long-acting insulin units | | 0.29 (0.24-0.36) |
| Total insulin units | | 0.64 (0.57-0.77) |
| Hemoglobin A1c (HbA1c), %, | | 8.05 (7.225 - 9.375) |
| Haemoglobin, g/L | | 136 (124-149.5) |
| Estimated glomerular filtration rate ml/min/1.73m^2^ | | 98.921 (79.288 - 111.175) |
| Metabolic Syndrome N (%) | | 36 (48%) |
| *Diabetes-related complications* | | |
| Hypertension, N (%) | | 44 (58.7%) |
| Retinopathy, N (%) | | 39 (52%) |
| Autoimmune thyroid disease, N (%) | | 22 (29.3%) |
| Cardiovascular disease, N (%) | | 14 (18.7%) |
| Kidney transplantation, N (%) | | 6 (8%) |
| Dialysis, N (%) | | 8 (10.7%) |
| Albuminuria classification | Normoalbuminuric | 40 (53.3%) |
|  | Microalbuminuria | 21 (28%) |
|  | Macroalbuminuria/ESRD | 14 (18.7%) |
| Albuminuria, mg/mmol | | 1.55 (0.557 - 6.593) |
| *Liver Function and MASLD Risk Markers* | | |
| Alanine transaminase (ALT), U/L | | 23 (18 - 30) |
| Aspartate aminotransferase, U/L | | 25.5 (19.25 - 32) |
| Hepatic steatosis index | | 35.725 (33.106 - 38.576) |
| Fatty liver index | | 17.952 (8.159 - 43.564) |
| Gamma-glutamyl transferase, U/L | | 16.5 (13 - 23.5) |
| *Inflammatory Markers* | | |
| C-reactive protein, mg/l | | 0.975 (0.552 - 2.12) |
| Lipopolysaccharide (LPS) concentration in serum, EU/ml | | 0.6 (0.515 - 0.679) |
| Procalcitonin concentration, ng/ml | | 2.837 (0.49 - 4.044) |
| Lipopolysaccharide-binding protein (LBP), EU/mL | | 9.621 (6.751 - 13.543) |
| EndoCAb Immunoglobulin G | | 58.994 (39.894 - 104.734) |
| EndoCAb Immunoglobulin M | | 30.456 (21.299 - 43.046) |
| LPS to HDL ratio | | 0.328 (0.254 - 0.443) |

Continuous variables are presented as medians (IQR).

**Supplementary table 2.** CGM parameters in whole cohort.

| **CGM Parameters** | **N =75** |
| --- | --- |
| Average glucose levels | 9.6 (8.1 - 11.35) |
| Coefficient of variance (CV) | 39.3 (35.65 - 42.4) |
| Glucose management indicator (GMI) % | 7.45 (6.825 - 8.2) |
| % time above range (TAR) | 43 (28 - 59) |
| % time in range (TIR) | 50 (37 - 65.25) |
| % time below range (TBR) | 4 (2 - 9.5) |
| Low glucose events | 9 (4 - 15) |
| Low glucose events average duration in min | 109 (76 - 131.5) |
| Estimated A1c% | 7.7 (6.725 - 8.7) |

Continuous variables are presented as medians (IQR).

**Supplementary table 3.** CGM parameter correlations with liver markers and inflammation markers in whole cohort

| Parameter 1 | Parameter 2 | Correlation Coefficient (95% CI) | p |
| --- | --- | --- | --- |
| Low glucose events | CRP | -0.34, (-0.53, -0.11) | 0.003 |
|  | LBP | -0.32, (-0.52, -0.1) | 0.005 |
|  | ALT | -0.25, (-0.45, -0.02) | 0.032 |
|  | HSI | -0.25, (-0.45, -0.02) | 0.032 |
|  | FLI | -0.28, (-0.48, -0.05) | 0.015 |
| % time above range (TAR) | CRP | 0.24, (0.01, 0.45) | 0.036 |
|  | LBP | 0.3, (0.07, 0.5) | 0.009 |
|  | GGT | 0.28, (0.05, 0.48) | 0.014 |
|  | ALT | 0.37, (0.14, 0.55) | 0.001 |
|  | AST | 0.23, (0, 0.43) | 0.049 |
|  | HSI | 0.27, (0.04, 0.47) | 0.02 |
|  | FLI | 0.3, (0.07, 0.5) | 0.009 |
| % time in range (TIR) | CRP | -0.28, (-0.48, -0.05) | 0.016 |
|  | LBP | -0.27, (-0.47, -0.04) | 0.021 |
|  | GGT | -0.29, (-0.49, -0.06) | 0.011 |
|  | ALT | -0.36, (-0.55, -0.14) | 0.001 |
|  | AST | -0.22, (-0.43, 0.01) | 0.053 |
|  | HSI | -0.28, (-0.48, -0.05) | 0.015 |
|  | FLI | -0.31, (-0.5, -0.08) | 0.007 |
| % time below range (TBR) | CRP | -0.25, (-0.46, -0.02) | 0.029 |
|  | LBP | -0.27, (-0.47, -0.04) | 0.019 |
|  | ALT | -0.22, (-0.43, 0.01) | 0.054 |
| Average glucose levels | CRP | 0.24, (0.01, 0.45) | 0.036 |
|  | LBP | 0.32, (0.09, 0.51) | 0.005 |
|  | GGT | 0.27, (0.04, 0.47) | 0.019 |
|  | ALT | 0.29, (0.07, 0.49) | 0.01 |
|  | FLI | 0.26, (0.03, 0.47) | 0.023 |
| Glucose management indicator (GMI) % | CRP | 0.24, (0.01, 0.45) | 0.036 |
|  | LBP | 0.32, (0.09, 0.51) | 0.005 |
|  | GGT | 0.27, (0.04, 0.47) | 0.019 |
|  | ALT | 0.29, (0.07, 0.49) | 0.01 |
|  | FLI | 0.26, (0.03, 0.47) | 0.023 |
| Estimated A1C | CRP | 0.24, (0.01, 0.45) | 0.035 |
|  | LBP | 0.31, (0.09, 0.51) | 0.006 |
|  | GGT | 0.27, (0.04, 0.47) | 0.019 |
|  | ALT | 0.3, (0.08, 0.5) | 0.008 |
|  | AST | 0.21, (-0.02, 0.42) | 0.075 |
|  | HSI | 0.21, (-0.02, 0.42) | 0.07 |
|  | FLI | 0.27, (0.04, 0.47) | 0.02 |

CRP - C-reactive protein, mg/l; LBP - Lipopolysaccharide-binding protein EU/mL; ALT - Alanine transaminase (ALT), U/L ; AST - Aspartate aminotransferase, U/L; GGT - Gamma-glutamyl transferase, U/L; FLI - Fatty liver index; HSI- Hepatic steatosis index.

**Supplementary table 4.** Inflammatory marker correlations with liver markers by cluster groups

| Parameter 1 | Parameter 2 | Cluster | Correlation Coefficient (95% CI) | p |
| --- | --- | --- | --- | --- |
| Lipopolysaccharide-binding protein (LBP), EU/mL | Fatty liver index | PCD | 0.39 ( 0.01 - 0.69 ) | 0.039 |
|  |  | MCD | 0.04(-0.25-0.32) | 0.802 |
|  | Hepatic steatosis index | PCD | 0.45 (0.08-0.72) | 0.016 |
|  |  | MCD | 0.02 (-0.27-0.31) | 0.880 |
|  | High-density lipoprotein cholesterol, mmol/l | PCD | -0.32 (-0.63--.07) | 0.094 |
|  |  | MCD | -0.27 (-0.52-0.02) | 0.063 |
| C-reactive protein, mg/l | Fatty liver index | PCD | 0.62 (0.29-0.82) | 0.000 |
|  |  | MCD | 0.16 (-0.14-0.43) | 0.286 |
|  | Hepatic steatosis index | PCD | 0.61 (0.28-0.81) | 0.001 |
|  |  | MCD | 0.15 (-0.14-0.42) | 0.310 |
|  | Low-density lipoprotein cholesterol, mmol/l | PCD | 0.08 (-0.30-0.44) | 0.690 |
|  |  | MCD | 0.36 (0.08-0.60) | 0.012 |
|  | Total cholesterol, mmol/l | PCD | 0.22 (-0.18-0.55) | 0.271 |
|  |  | MCD | 0.36 (0.07-0.59) | 0.013 |
| Procalcitonin concentration, ng/ml | Hepatic steatosis index | PCD | 0.59 (0.25-0.80) | 0.001 |
|  |  | MCD | 0.34 (0.05-0.58) | 0.019 |
|  | Aspartate aminotransferase U/L | PCD | -0.13 (-0.48-0.26) | 0.519 |
|  |  | MCD | -0.30 (-0.55-0.01) | 0.039 |
|  | Alanine transaminase U/L | PCD | -0.10 (-0.46-0.28) | 0.612 |
|  |  | MCD | -0.25 (-0.51-0.04) | 0.088 |
| Lipopolysaccharide (LPS) concentration in serum, EU/ml | Fatty liver index | PCD | 0.430 (0.050-0.701) | 0.023 |
|  |  | MCD | 0.35 (0.06-0.59) | 0.015 |
|  | High-density lipoprotein cholesterol, mmol/l | PCD | -0.507 (-0.751-; -0.141) | 0.006 |
|  |  | MCD | -0.26 (-0.52-0.03) | 0.073 |
|  | Triglycerides mmol/l | PCD | 0.659 (0.344-0.841) | 0.000 |
|  |  | MCD | 0.62 (0.38-0.78) | 0.000 |
|  | Aspartate aminotransferase U/L | PCD | -0.013 (-0.384-0.362) | 0.949 |
|  |  | MCD | 0.29 (-0.01-0.53) | 0.051 |
|  | Estimated glucose disposal rate mg/kg/min | PCD | -0.278 (-0.595-0.151) | 0.151 |
|  |  | MCD | -0.42 (-0.64-0.14) | 0.003 |
| EndoCab Immunoglobulin M | High-density lipoprotein cholesterol, mmol/l | PCD | 0.39 (0.01-0.67) | 0.040 |
|  |  | MCD | 0.08 (-0.21-0.36) | 0.594 |
|  | Low-density lipoprotein cholesterol, mmol/l | PCD | 0.33 (-0.06-0.63) | 0.091 |
|  |  | MCD | -0.17 (-0.44-0.12) | 0.248 |
|  | Total cholesterol, mmol/l | PCD | 0.45 (0.08-0.72) | 0.016 |
|  |  | MCD | -0.08 (-0.36-0.22) | 0.609 |
|  | Triglycerides, mmol/l, | PCD | -0.36 (-0.65-0.03) | 0.062 |
|  |  | MCD | -0.04 (-0.33-0.25) | 0.779 |
|  | Alanine transaminase U/L | PCD | -0.46 (-0.72- -0.08) | 0.014 |
|  |  | MCD | -0.12 (-0.40-0.17) | 0.416 |
| EndoCab Immunoglobulin G | High-density lipoprotein cholesterol, mmol/l | PCD | 0.34 (-0.05-0.64) | 0.074 |
|  |  | MCD | -0.07 (-0.35-0.23) | 0.659 |
|  | Triglycerides, mmol/l | PCD | -0.58 (-0.80 -; -0.24) | 0.001 |
|  |  | MCD | 0.26 (-0.03 - 0.51) | 0.075 |
|  | Alanine transaminase U/L | PCD | -0.38 (-0.67-0.01) | 0.048 |
|  |  | MCD | -0.17 (-0.44-0.13) | 0.260 |
|  | Estimated glucose disposal rate mg/kg/min | PCD | 0.38 (0.00-0.67) | 0.045 |
|  |  | MCD | -0.01 (-0.30-0.28) | 0.933 |

PCD – poorly controlled diabetes; MCD – Moderately controlled diabetes

**Supplementary Table 5.** Chi-square test results, fatty liver index (FLI), and hepatic steatosis index (HSI) by study group.

| Index | Group (cutoff) | Poorly CD | Moderately CD | P |
| --- | --- | --- | --- | --- |
| Hepatic steatosis index by 2 | <36 ruling out or indeterminate) | 10 (35.714%) | 28 (59.574%) | 0.046 |
|  | ≥36 (ruling in steatosis) | 18 (64.286%) | 19 (40.426%) |  |
| Fatty liver index by 2 | <60 ruling out or indeterminate | 18 (64.286%) | 42 (89.362%) | 0.009 |
|  | ≥60 ruling in steatosis | 10 (35.714%) | 5 (10.638%) |  |

**Supplementary Table 6.**Full exploratory mediation analysis results for 12 tested models.

| **Mediation model number in manuscript** | **Mediator** | **Outcome** | **ACME (*a***×***b*)** | **ADE (*c’*)** | **Total effect (*c’+a***×***b*)** |
| --- | --- | --- | --- | --- | --- |
| I | FLI  *a*=-0.62, *p*=0.008  *a*=-0.55,*p*=0.013 | LBP  *b*=0.22, *p*=0.061  *b*=0.34, *p*=0.008 | -0.14 (-0.37, 0.02), *p*=0.098  -0.19 (-0.44, 0.01), *p*=0.031 | -0.40 (-0.90, 0.08), *p*=0.098  -0.35 (-0.85, 0.10), *p*=0.13 | -0.53 (-1.03, -0.06), *p*=0.026  -0.54 (-1.07, -0.05), *p*=0.028 |
| II |  | CRP  *b*=0.32, *p*=0.009  *b*=0.42, *p*=0.001 | -0.20 (-0.54, -0.01), *p*=0.029  -0.24 (-0.60, -0.02), *p*=0.020 | 0.117 (-0.41, 0.76), *p*=0.79  0.18 (-0.35, 0.81), *p*=0.65 | -0.08 (-0.47, 0.36), *p*=0.66  -0.06 (-0.46, 0.39), *p*=0.74 |
| III |  | Endo Cab IgM  *b*=-0.24, *p*=0.053  *b*=-0.15, *p*=0.24 | 0.15 (0.01, 0.38), *p*=0.036  0.09 (-0.03, 0.28), *p*=0.18 | -0.11 (-0.63, 0.38), *p*=0.70  -0.06 (-0.57, 0.41), *p*=0.83 | 0.04 (-0.46, 0.51), *p*=0.82  0.03 (-0.44, 0.47), *p*=0.88 |
| IV |  | LPS  *b*=0.22, *p*=0.071  *b*=0.14, *p*=0.30 | -0.14 (-0.36, -0.01), *p*=0.023  -0.07 (-0.29, 0.07), *p*=0.33 | -0.005 (-0.38, 0.44), *p*=0.96  -0.03 (-0.41, 0.39), *p*=0.85 | -0.14 (-0.54, 0.29), *p*=0.47  -0.10 (-0.55, 0.37), *p*=0.61 |
| VII |  | Endo Cab IgG  *b*=-0.12, *p*=0.31  *b*=-0.11, *p*=0.38 | 0.08 (-0.03, 0.22), *p*=0.16  0.06 (-0.04, 0.19), *p*=0.23 | 0.05 (-0.36, 0.51), *p*=0.80  0.15 (-0.29, 0.64), *p*=0.51 | 0.13 (-0.28, 0.56), *p*=0.55  0.22 (-0.21, 0.69), *p*=0.34 |
| VIII |  | ProC  *b*=0.17, *p*=0.17  *b*=0.34, *p*=0.003 | -0.11 (-0.31, 0.03), *p*=0.15  -0.19 (-0.40, -0.02), *p*=0.02 | 0.17 (-0.30, 0.64), *p*=0.49  0.32 (-0.11, 0.72), *p*=0.13 | 0.06 (-0.39, 0.50), *p*=0.77  0.13 (-0.27, 0.52), *p*=0.52 |
| V | HSI  *a* =-0.42, *p*=0.081  *a*=-0.36, *p*=0.14 | LBP  *b*=0.19, *p*=0.103  *b*=0.21, *p*=0.073 | -0.08 (-0.24, 0.02), *p*=0.16  -0.08 (-0.23, 0.03), *p*=0.18 | -0.45 (-0.97, 0.02), *p*=0.064  -0.46 (-1.00, 0.01), *p*=0.058 | -0.53 (-1.03, -0.06), *p*=0.026  -0.54 (-1.07, -0.05), *p*=0.028 |
| VI |  | ProC  *b*=0.34, *p*=0.004  *b*=0.36, *p*=0.001 | -0.14 (-0.42, 0.01), *p*=0.076  -0.13 (-0.38, 0.03), *p*=0.12 | 0.21 (-0.22, 0.67), *p*=0.33  0.26 (-0.13, 0.66), *p*=0.19 | 0.06 (-0.39, 0.50), *p*=0.77  0.13 (-0.27, 0.52), *p*=0.52 |
| IX |  | CRP  *b*=0.24, *p*=0.048  *b*=0.25, *p*=0.042 | -0.10 (-0.33, 0.02), *p*=0.11  -0.09 (-0.31, 0.03), *p*=0.16 | 0.02 (-0.44, 0.56), *p*=0.98  0.03 (-0.42, 0.56), *p*=0.98 | -0.08 (-0.47, 0.36), *p*=0.66  -0.06 (-0.46, 0.39), *p*=0.74 |
| X |  | Endo Cab IgM  *b*=-0.01, *p*=0.91  *b*=0.01, *p*=0.92 | 0.01 (-0.11, 0.11), *p*=0.92  -0.004 (-0.10, 0.09), *p*=0.94 | 0.04 (-0.46, 0.51), *p*=0.84  0.03 (-0.43, 0.48), *p*=0.87 | 0.04 (-0.46, 0.51), *p*=0.82  0.03 (-0.44, 0.47), *p*=0.88 |
| XI |  | Endo Cab IgG  *b*=-0.04, *p*=0.76  *b*=-0.06, *p*=0.61 | 0.02 (-0.09, 0.11), *p*=0.72  0.02 (-0.05, 0.12), *p*=0.58 | 0.11 (-0.30, 0.57), *p*=0.59  0.20 (-0.23, 0.67), *p*=0.39 | 0.13 (-0.28, 0.56), *p*=0.55  0.22 (-0.21, 0.69), *p*=0.34 |
| XII |  | LPS  *b*=-0.07, *p*=0.57  *b*=-0.11, *p*=0.38 | 0.03 (-0.11, 0.19), *p*=0.70  0.04 (-0.09, 0.20), *p*=0.61 | -0.17 (-0.52, 0.21), *p*=0.36  -0.14 (-0.55, 0.29), *p*=0.48 | -0.14 (-0.54, 0.29), *p*=0.47  -0.10 (-0.55, 0.37), *p*=0.61 |

Mediation analysis models were constructed for exploratory purposes using T1D control status as the exposure variable, MASLD indices as mediators, and endotoxaemia and systemic inflammation markers as outcome variables (see also mediation diagrams for Models I and V in Figure 5 as examples). Results are presented as estimates of path coefficients *a* and *b* from linear regression models, average causal mediation effects (ACME), average direct effects (ADE), and total effects with corresponding 95% confidence intervals. Mediation analyses were performed using the R package *mediation* with 5,000 bootstrap resamples and a fixed random seed. Models highlighted in red are not presented in the manuscript because no significant effects were observed. Results shown in the second line (blue) represent the same models after adjustment for age and sex. FLI - fatty liver index; HIS - hepatic steatosis index; LBP - lipopolysaccharide-binding protein; LPS - lipopolysaccharides; CRP - C-reactive protein; EndoCAb IgM - Endotoxin Core Antibody immunoglobulin M;EndoCAb IgM; ProC - procalcitonin.


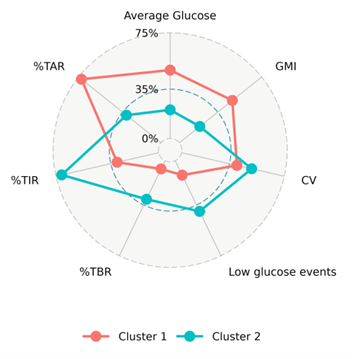


**Supplemental Figure 1.** Radar chart depicting the average percentage levels of seven continuous glucose monitoring (CGM) metrics: average glucose, glucose management indicator (GMI), coefficient of variation (CV), number of low glucose events, and the percentage of time spent in range (%TIR), below range (%TBR), and above range (%TAR) in two patient clusters—poorly controlled diabetes (Cluster 1) and moderately controlled diabetes (Cluster 2).


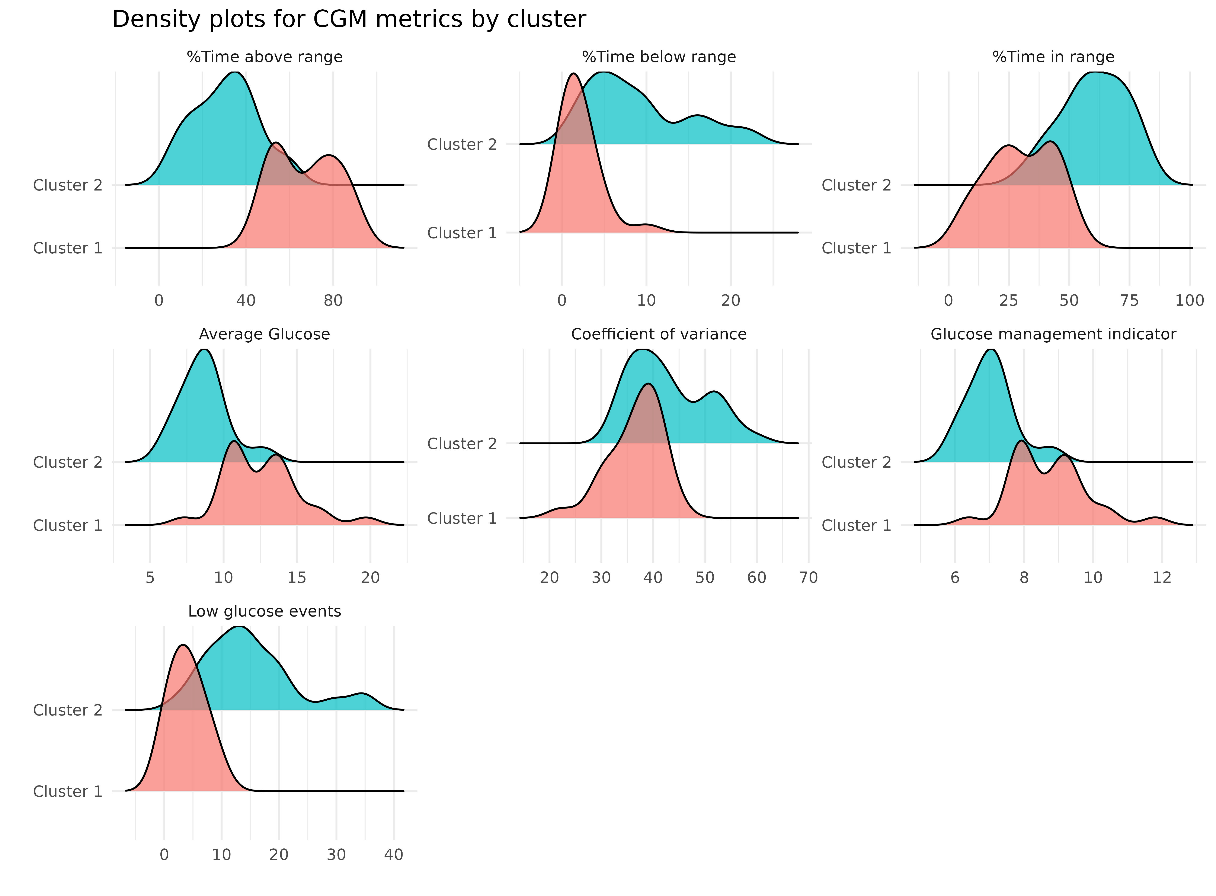


**Supplemental Figure 2.** Comparisons of data distributions shown with smoothed histograms for seven continuous glucose monitoring (CGM) metrics: average glucose, glucose management indicator (GMI), coefficient of variation (CV), number of low glucose events, and the percentage of time spent in range (%TIR), below range (%TBR), and above range (%TAR), across two patient clusters—poorly controlled diabetes (Cluster 1) and moderately controlled diabetes (Cluster 2).
